# Supplementary figures and images for: Connection between the Gut Microbiota of Largemouth Bass (Micropterus salmoides) and Microbiota of the Pond Culture Environment
Source: Microorganisms. 2021 Aug 19;9(8):1770. doi: 10.3390/microorganisms9081770 (PMC8402112; doi:10.3390/microorganisms9081770)

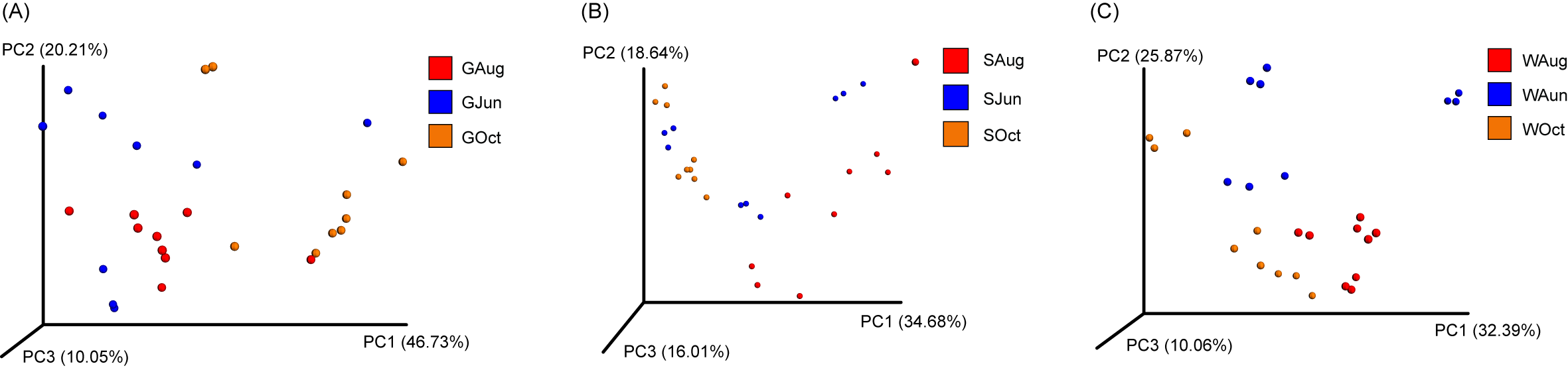

Supplement: Supplementary file 1 [file microorganisms-09-01770-s001.zip › Figure_S2.tif]

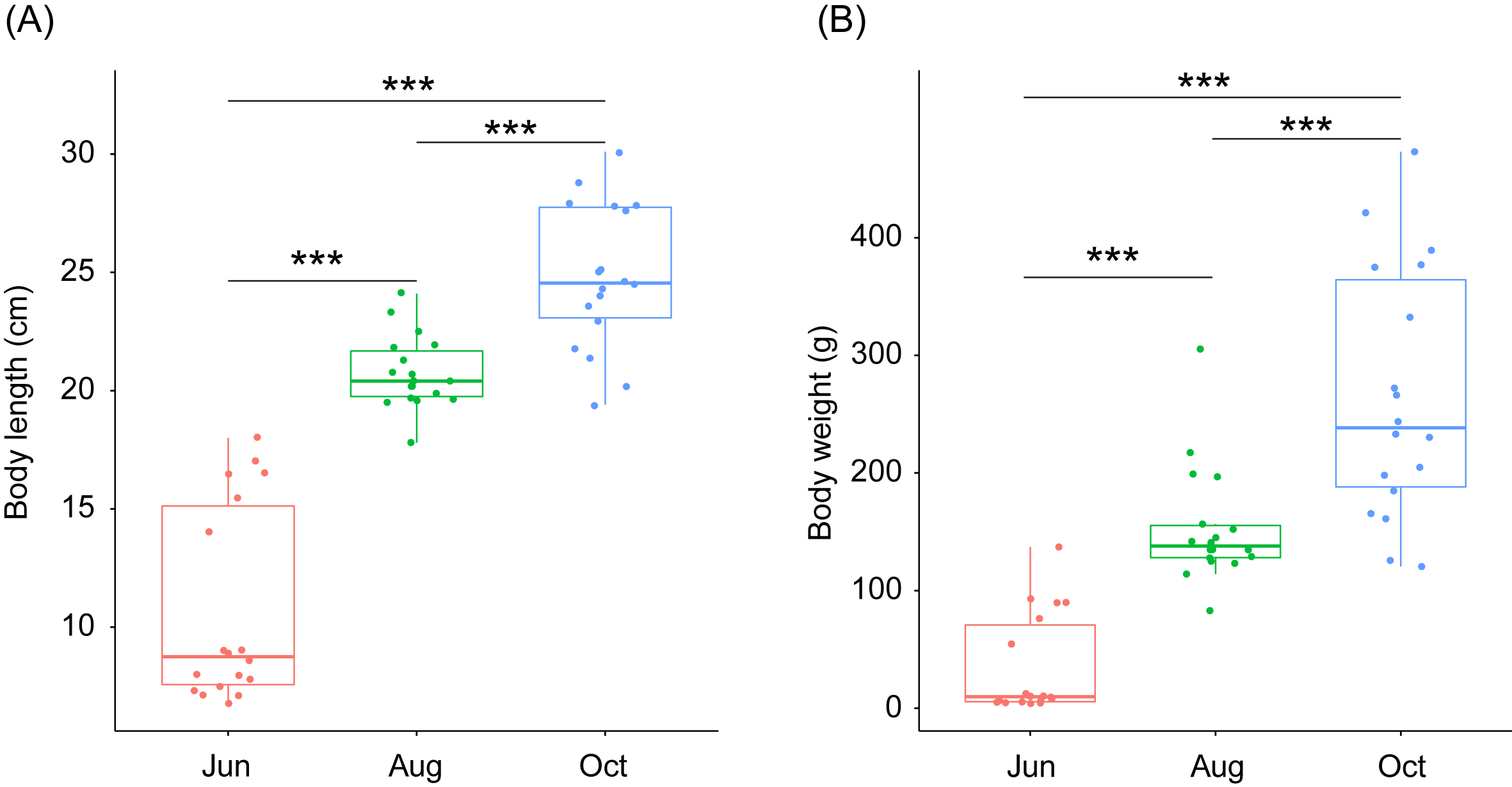

Supplement: Supplementary file 1 [file microorganisms-09-01770-s001.zip › Figure_S1.tif]
